# A case report of cardiac neuromodulation in a young patient with a third-degree atrioventricular block

**DOI:** 10.3389/fcvm.2024.1370522

**Published:** 2024-04-03

**Authors:** Noemi Valenti, Antonio Di Monaco, Imma Romanazzi, Nicola Vitulano, Federica Troisi, Federico Quadrini, Massimo Grimaldi

**Affiliations:** Department of Cardiology, General Regional Hospital “F. Miulli”, Acquaviva delle Fonti, Bari, Italy

**Keywords:** autonomic nervous system (ANS), cardioneuroablation (CNA), catheter ablation, case report, third-degree atrioventricular block

## Abstract

**Background:**

There are some functional bradyarrhythmias that are caused by a dysregulation of the autonomic nervous system, for which a therapeutic strategy of cardioneuroablation (CNA) is conceivable.

**Case summary:**

In this study, we report the case of a 19-year-old woman with a non-congenital third-degree atrioventricular block (AVB), symptomatic for lipothymia and dyspnea caused by mild exertion. She had a structurally normal heart and no other comorbidities. The atropine test and the exercise stress test documented a sinus tachycardia at 190 bpm with a 2:1 AVB, a narrow QRS, and an atrioventricular conduction of 1:1 until reaching a sinus rhythm rate of 90 bpm. She underwent the CNA procedure, which targeted the inferior paraseptal ganglion plexus, with a gradual change in the ECG levels recorded during the radiofrequency delivery from a third-degree AVB to a first-degree AVB. After the procedure, we observed a complete regression of the third-degree AVB, with evidence of only a first-degree AVB and a complete regression of symptoms until the 6-month follow-up.

**Conclusions:**

Although not yet included in current guidelines, the CNA procedure could be used to treat AV node dysfunction in young subjects, as it could represent an alternative to pacemaker implantation. However, more randomized studies are needed to assess the long-term efficacy of this promising technique.

## Introduction

The autonomic nervous system plays an important role in modulating the cardiovascular system. However, there are some pathological conditions that are caused by dysregulation of this system, particularly hyperactivity of the parasympathetic component, for which a therapeutic strategy of ablation of the cardiac paraganglia from the atrial endocardial side (cardioneuroablation, or CNA) is conceivable. According to the most recent evidence, in patients with reflex syncope, the CNA procedure is able to prevent syncopal recurrences, with an increase in the heart rate and a reduction in its variability at least during the first 2 years after the procedure (1–4).

## Clinical description

**Figure 1:**
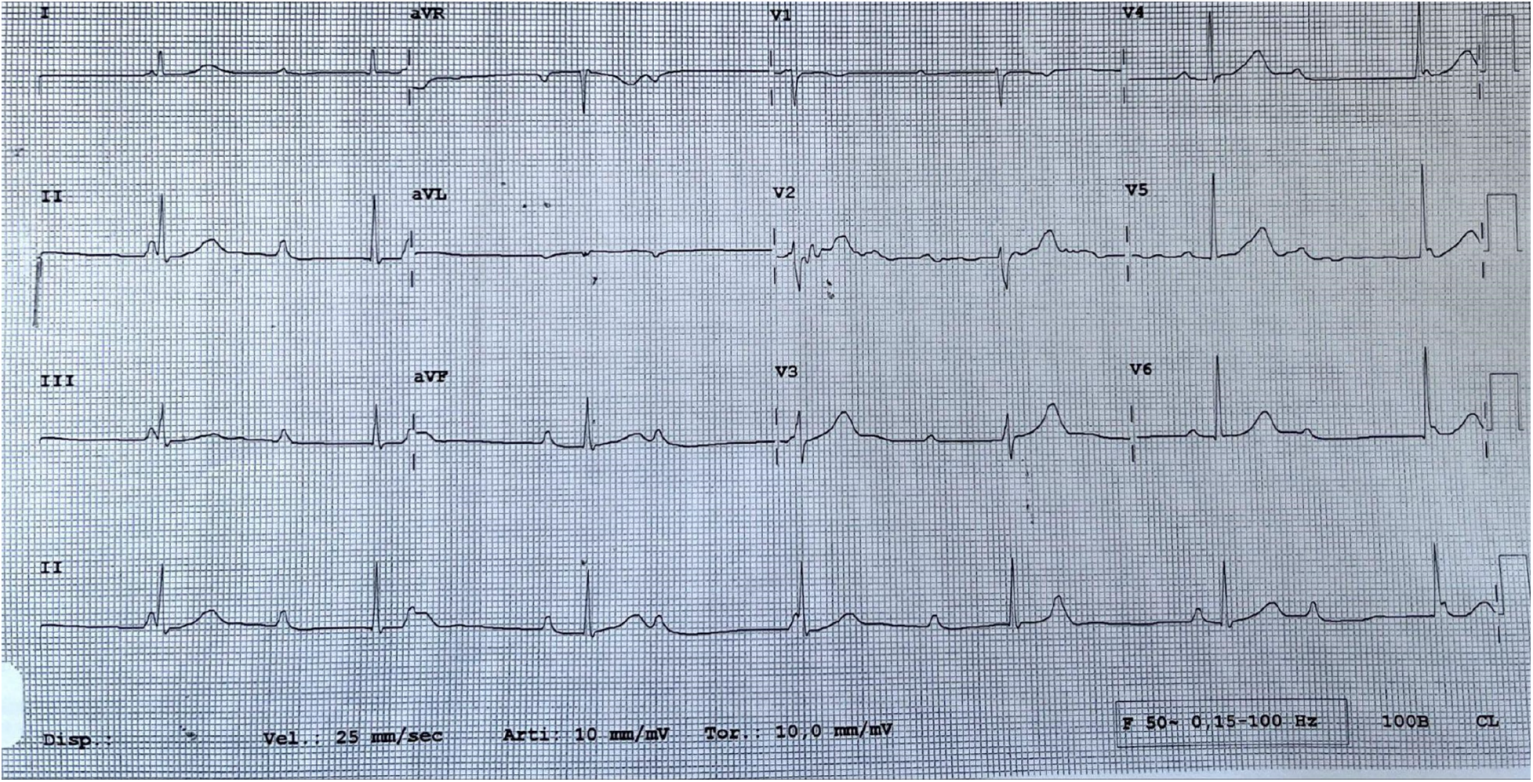
A third-degree atrioventricular block before the CNA procedure.

A 19-year-old woman was admitted to our hospital with a third-degree atrioventricular block (AVB) (Figure 1), symptomatic for lipothymia and dyspnea caused by mild exertion. The patient was not affected by a congenital third-degree AVB, and at the age of 13 years, her ECG showed only a first-degree AVB. Over the years, the PQ interval progressively lengthened until it became a fixed third-degree atrioventricular block. Cardiac magnetic resonance documented a structurally normal heart; the patient had no other comorbidities and was not taking any medications. The woman had no family history of cardiac conduction system disorders, cardiomyopathy, sudden death, isolated early-onset atrial fibrillation, or any other electrical disorder. The results of her genetic analysis did not identify any mutations possibly related to the conduction disorder.

The 24-h ECG Holter monitoring reported a persistent third-degree AVB with a narrow QRS escape rhythm at a mean heart rate of 40 bpm. The exercise stress test documented, at the maximum effort, a sinus tachycardia at 196 bpm with a 2:1 AVB and a narrow QRS. The atropine test (at a dose of 0.04 mg/kg) showed sinus tachycardia at 190 bpm with a 2:1 AVB and a narrow QRS. During the performance of the effort and atropine tests, the atrioventricular conduction was 1:1 until reaching a sinus rhythm rate of 90 bpm. These changes were demonstrative of the dominant functional component underlying the disorder.

In particular, at the beginning of the atropine test, the sinus rate was 80 bpm, and the patient had a third-degree AVB. Atropine initially caused a change in the AVB from a third-degree block to a first-degree one, and when the atropine also affected the sinus node with an increase in the sinus rate, the degree of the AVB worsened to a 2:1 atrioventricular conduction, probably because the Luciani–Wenckebach point was exceeded. For this reason, to avoid an excessive increase in the sinus node rate, we decided to perform a selective cardioneuroablation procedure for the atrioventricular node.

**Figure 2:**
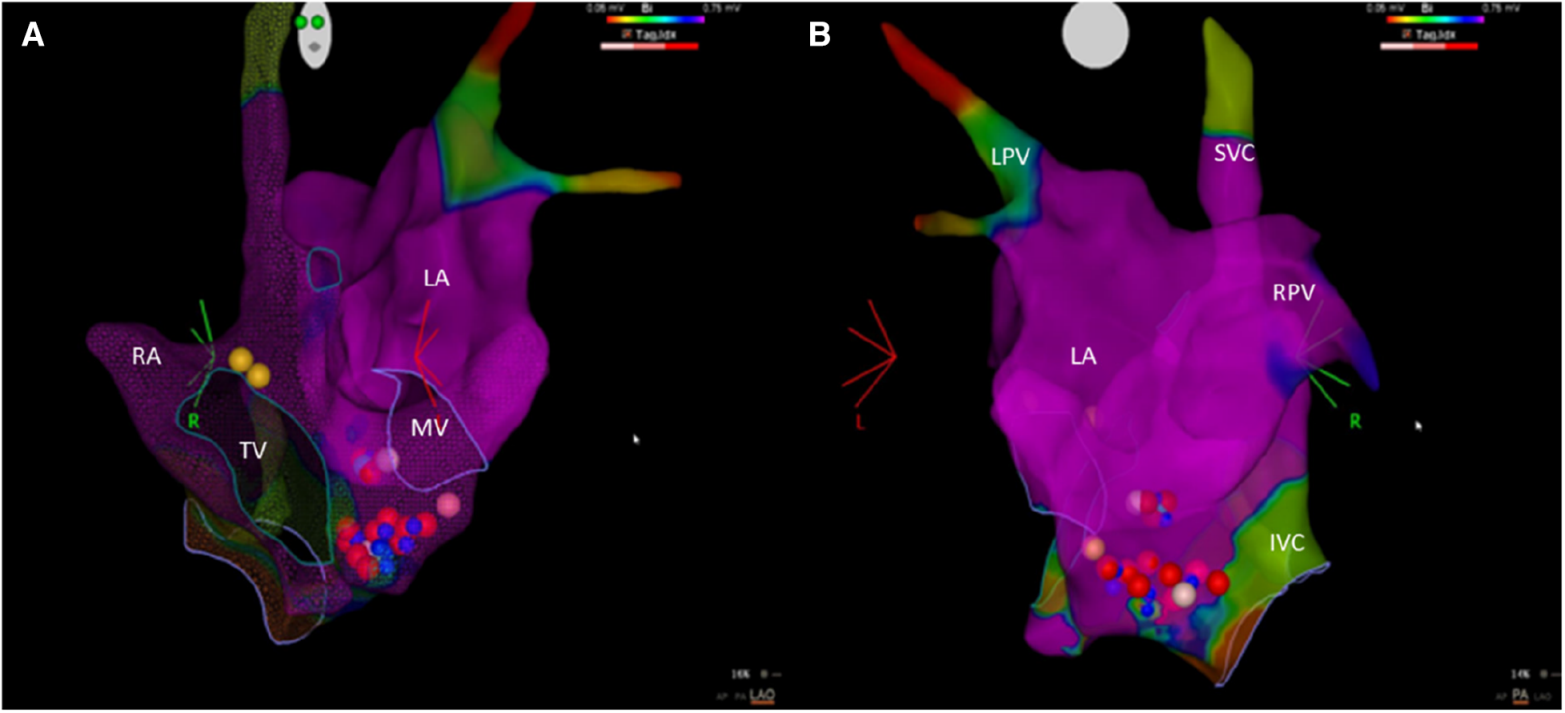
An electro-anatomic mapping of the left and right atria using the CARTO system. (**A**) An anterior–posterior view. (**B**) A left-lateral view. The blue dots are regions where radiofrequency delivery caused a slowing of the narrow QRS escape rhythm. The red and pink dots are the points of ablation with an ablation index of 550 or <550, respectively. The yellow dots indicate the location of the bundle of His. IVC, inferior vena cava; LA, left atrium; LPV, left pulmonary veins; MV, mitral valve; RA, right atrium; RPV, right pulmonary veins; SVC, superior vena cava; TV, tricuspid valve.

With the aim of postponing the implantation of a definitive pacemaker, a procedure rejected by the patient and her family, we performed the CNA procedure on the patient after providing adequate information on this procedure to her and after she accepted the intervention. The procedure was performed under light sedation with diazepam. An electrophysiological examination showed impaired atrioventricular nodal conduction, but His-Purkinje conduction was preserved (normal HV interval). The ablation procedure targeted the inferior paraseptal ganglion plexus (IPSGP), located in the posteroinferior area of the right atrial septum, close to the proximal coronary sinus (and around its ostium), the structure mainly responsible for the parasympathetic innervation of the atrioventricular node. The ablation was performed from both the right (10 radiofrequency deliveries) and the left atrial sides, thereby also affecting the component of the IPSGP known in the literature as the posteromedial left atrial ganglionated plexus (PMLGP). The ablation catheter Q-DOT® (Biosense Webster, Diamond Bar, CA, USA) was anatomically positioned on the IPSGP area (from both the right and left atrial sides), and radiofrequency was delivered. For each application, the power used was 35 W for 60 s, obtaining an ablation index (a parameter that takes into consideration the power, time, and stability of the lesion) between 500 and 550 (Figure 2). We administered heparin to the patient in order to maintain an activated clotting time >300 s. The radiofrequency applications delivered in the right atrium did not change the baseline ECG level, whereas the applications in the left atrium gradually changed the ECG level from a third-degree AVB to a first-degree one. We decided to stop the radiofrequency applications in the left atrium as soon as the third-degree AV block resolved, maintaining only a first-degree AV block with a stable PQ that showed no signs of fluctuation. The Wenckebach point and the AH interval post-cardioneuroablation were 450 and 220 ms, respectively. No procedural or post-procedural complications occurred.

After the procedure, we observed a complete regression of the third-degree AVB, with evidence of only a first-degree AVB (PQ interval 280 ms). An ECG Holter monitoring after the procedure recorded short nightly periods of a second-degree AVB (Mobitz 1 and rare 2).

**Figure 3:**
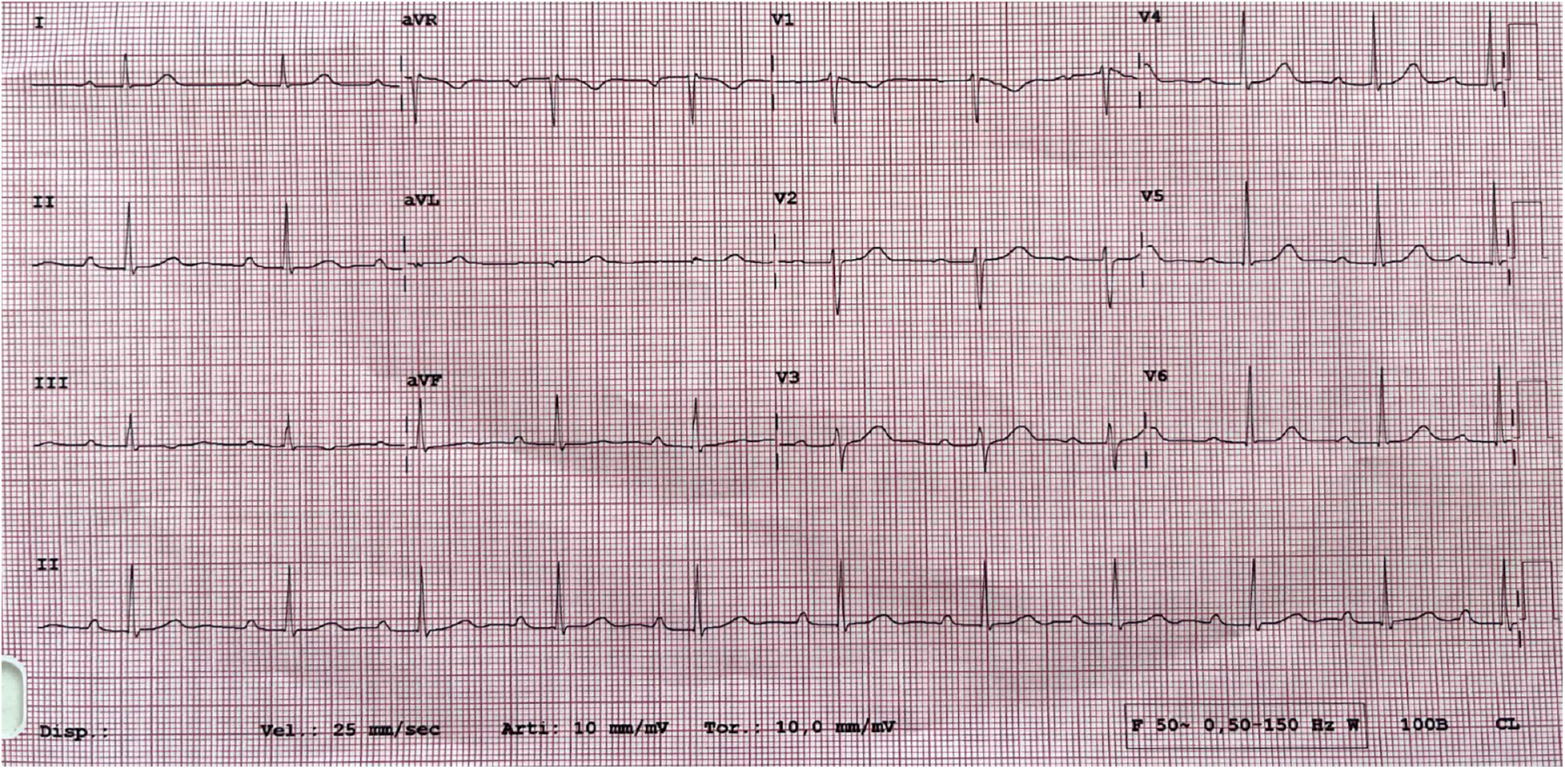
A 1-month follow-up ECG with a first-degree atrioventricular block (PQ interval 260 ms).

At 1-month post-procedure, there was a complete regression of symptoms and further reduction of the PQ interval (260 ms) on baseline ECG (Figure 3). The 24-h ECG Holter monitoring reported a short nightly episode of a second-degree AVB (Mobitz 2). During the performance of the exercise stress test, the atrioventricular conduction was 1:1 until reaching a sinus tachycardia of 151 bpm; the atrioventricular conduction was 2:1 when the sinus tachycardia was between 150 and 170 bpm.

At 6 months’ post-procedure, the patient reported total asymptomaticity with a clear improvement in her quality of life; the ECG and 24-h-Holter results appeared unchanged compared with the results of the tests performed 1 month after the CNA procedure. To date, no QTc prolongation, ventricular arrhythmias, or inappropriate sinus tachycardia have occurred.

## Discussion

In the last decade, the importance of the autonomic nervous system has been highlighted for enabling a better understanding of the several mechanisms involved in the development of cardiac arrhythmias, especially atrial fibrillation.

The beat-to-beat information is detected by cardiac afferents (chemoreceptors and mechanoreceptors), transduced centrally, and reprocessed in a series of interacting feedback loops to modulate the efferent flow in the form of parasympathetic output through the vagus nerve and sympathetic output through the extracardiac intrathoracic ganglia. At the cardiac level, the structures responsible for maintaining these connections are the ganglionated plexuses (GPs), which are incorporated into the epicardial fat pads and in the heart muscle, mainly on the posterior side of the atria. GPs serve as integrating centers for afferent information and efferent outflow to regulate cardiac electrical and contractile functions. They contain afferent neurons, efferent neurons (sympathetic and parasympathetic), and interconnected local circuit neurons, but with a good percentage of parasympathetic neurons, which are precisely the target of the CNA procedure (5). Therefore, since the complex intracardiac nervous system, together with the extracardiac autonomic nervous system, plays a fundamental role in the regulation of cardiac activity in both physiological and pathological conditions, a modulation strategy of the key neural connections to the heart using endocardial ablation is conceivable.

The main locations of the GPs include the following: the superior paraseptal ganglionated plexus (SPSGP, located in the upper area of the right atrium, medial to the outlet of the superior vena cava and lateral to the aortic root) which is the final common pathway of the right vagus that innervates the sinus node; the aorta-superior vena cava ganglionated plexus (Ao-SVC GP), anterior to the previous one; the inferior paraseptal ganglionated plexus (IPSGP, located in the posteroinferior area of the right interatrial septum, near the proximal coronary sinus, and around its ostium) which is the final common pathway of the left vagus that innervates the AV node; the left superior ganglionated plexus (LSGP); the left lateral ganglionated plexus (LLGP), also called GP of Marshall’s ligament; right and left inferior ganglionated plexuses (RIGP and LIGP), sometimes identified as oblique sinus GP (OSGP) (1, 5–11).

The majority of the mentioned localizations of the GPs are already potential targets during catheter ablation of atrial fibrillation, but nowadays, it is possible to perform additional radiofrequency applications with the aim of obtaining neuromodulation of the sinus node or atrioventricular node in appropriately selected patients, even outside the atrial fibrillation ablation procedure.

In the patient case described in this study, the ablation affected the IPSGP, both from the right and left atrial sides, without extending to the further paraganglionic stations. The logic of our choice is related both to the fact that during the ablation of this paraganglion alone, we obtained a sustained 1:1 atrioventricular conduction and to the fact that in existing studies on the subject, the ablation of this GP was the one that demonstrated the greatest effects on atrioventricular conduction. The extension of the lesions also to the stations innervating mainly the sinus node could lead to a greater risk of inappropriate sinus tachycardia and paradoxically worsen the ability of the atrioventricular node to conduct sinus impulses of 1:1.

At present, the majority of CNA procedures are being performed in patients with neuromediated cardioinhibitory syncope and then in patients with functional bradyarrhythmias. There is limited data on the use of the CNA procedure in the context of a functional atrioventricular block (FAVB), especially in clinical cases or case series (12, 13). These patients, even without any organic damage to the conduction system, often present disabling symptoms with a significant reduction in the quality of life; treatment with drugs and/or pacemakers provides poor results. Furthermore, pacemaker implantation is not well accepted and is accompanied by significant psychological discomfort, in addition to a risk of infection and long-term ventricular desynchronization. The absence of an organic disease, as well as a presumed benign course of the disease, makes the decision to carry out implantation more difficult (12). Therefore, a strategy like the one described in this study could represent a solution to the problem.

Furthermore, another important question is the lack of a standardized procedure to perform CNA. It is not known which of the following is the best approach to identify target sites for ablation: an exclusively anatomical approach using a three-dimensional endocardial electro-anatomical mapping system; detection of a vagal response in response to high-frequency stimulation (HFS); spectral analysis of atrial potentials; identification of fractionated atrial electrograms; contrast computed tomography (CT) or single photon emission computed tomography (SPECT) (1). Because no comparative data are available on these methods, the optimal GP mapping technique to prevent re-innervation and achieve long-term clinical benefit has yet to be determined (14). Furthermore, a recent meta-analysis of cardioneuroablation comparing different methods of locating target sites for GP ablation found no differences in GP analysis subgroups (4).

Moreover, it is not clear whether the ablation procedure must be performed in the right or left atrium or both, and there is no sufficient data regarding the predictors of procedural efficacy.

Also, the long-term results of the CNA procedure are not well known, and some data in the scientific literature have revealed a possible re-innervation of the structures after ablation (not necessarily associated with a recurrence of symptoms). The risk of re-innervation would appear to be related to both procedural factors (ablative lesions not deep enough to reach the epicardium or non-appropriate targeting of ganglion sites) and patient-related factors (myocardial thickness and epicardial fat in critical regions, intra-individual variable capacity for neural regeneration) (1).

Given the absence of standardized approaches and large randomized studies in support of cardioneuroablation, this procedure should currently be considered an individualized treatment option to be used in selected patients with a dominant functional component underlying the bradyarrhythmic disorder. In such contexts, a strategy of modulation (with inhibition) of the paraganglia is also conceivable, rather than their ablation, using a non-invasive and temporary strategy (such as a low-level transcutaneous vagus nerve stimulation at the auricular level) with the aim of preserving the structural integrity of the intrinsic cardiac nervous system (ICNS) (15).

## Conclusions

Current guidelines recommend the implantation of a definitive pacemaker in patients with a paroxysmal or permanent third-degree AVB, regardless of symptoms (16). Although not yet included in current guidelines, the CNA procedure could be used to treat atrioventricular node dysfunction in young subjects with a dominant functional component, as it could represent an alternative to pacemaker implantation. Pacemaker implantation in young patients is associated with the risks of infection and malfunction over time, which reduces the quality of life.

More randomized studies are needed to assess the long-term efficacy of the promising CNA technique.

## Data Availability

The raw data supporting the conclusions of this article will be made available by the authors, without undue reservation.

## References

[1] Brignole M Aksu T Calò L Debruyne P Deharo JC Fanciulli A Clinical controversy: methodology and indications of cardioneuroablation for reflex syncope. Europace. (2023) 25(5):euad033. 10.1093/europace/euad033 37021351 PMC10227654

[2] Soni B Gupta D Gopinathannair R. Quality of life improvement following cardioneuroablation for vasovagal syncope: expected or too early to say? J Interv Card Electrophysiol. (2023). 10.1007/s10840-023-01489-w. [Epub ahead of print] 36705871

[3] Piotrowski R Baran J Sikorska A Krynski T Kulakowski P. Cardioneuroablation for reflex syncope: efficacy and effects on autonomic cardiac regulation—a prospective randomized trial. JACC Clin Electrophysiol. (2023) 9(1):85–95. 10.1016/j.jacep.2022.08.011 36114133

[4] Vandenberk B Lei LY Ballantyne B Vickers D Liang Z Sheldon RS Cardioneuroablation for vasovagal syncope: a systematic review and meta-analysis. Heart Rhythm. (2022) 19(11):1804–12. 10.1016/j.hrthm.2022.06.017 35716859

[5] Hanna P Buch E Stavrakis S Meyer C Tompkins JD Ardell JL Neuroscientific therapies for atrial fibrillation. Cardiovasc Res. (2021) 117(7):1732–45. 10.1093/cvr/cvab172 33989382 PMC8208752

[6] Santos Silva G Fonseca P Cardoso F Almeida J Ribeiro S Oliveira M Cardioneuroablation for severe neurocardiogenic syncope. Rev Port Cardiol. (2023) 42(10):821–9. 10.1016/j.repc.2023.02.012 37268266

[7] Francia P Viveros D Falasconi G Soto-Iglesias D Fernández-Armenta J Penela D Computed tomography-based identification of ganglionated plexi to guide cardioneuroablation for vasovagal syncope. Europace. (2023) 25(6):euad170. 10.1093/europace/euad170 37343139 PMC10311406

[8] Aksu T Skeete JR Huang HH. Ganglionic plexus ablation: a step-by-step guide for electrophysiologists and review of modalities for neuromodulation for the management of atrial fibrillation. Arrhythm Electrophysiol Rev. (2023) 12:e02. 10.15420/aer.2022.37 36845167 PMC9945432

[9] Li L Po S Yao Y. Cardioneuroablation for treating vasovagal syncope: current status and future directions. Arrhythm Electrophysiol Rev. (2023) 12:e18. 10.15420/aer.2023.02 37457436 PMC10345939

[10] O’Brien B Reilly J Coffey K González-Suárez A Quinlan L van Zyl M. Cardioneuroablation using epicardial pulsed field ablation for the treatment of atrial fibrillation. J Cardiovas. Dev Dis. (2023) 10(6):238. 10.3390/jcdd10060238 PMC10299113 37367403

[11] Francia P Viveros D Falasconi G Penela D Soto-Iglesias D Martí-Almor J Clinical impact of aging on outcomes of cardioneuroablation for reflex syncope or functional bradycardia: results from the cardionEuroabLation: patiEnt selection, imaGe integrAtioN and outComEs—the ELEGANCE multicenter study. Heart Rhythm. (2023) 20(9):1279–86. 10.1016/j.hrthm.2023.06.007 37329936

[12] Pachon MJC Pachon MEI Lobo TJ Pachon MJC Pachon MZ Vargas RN Syncopal high-degree AV block treated with catheter RF ablation without pacemaker implantation. Pacing Clin Electrophysiol. (2006) 29(3):318–22. 10.1111/j.1540-8159.2006.00340.x 16606401

[13] Aksu T Gopinathannair R Bozyel S Yalin K Gupta D. Cardioneuroablation for treatment of atrioventricular block. Circ Arrhythm Electrophysiol. (2021) 14(9):e010018. 10.1161/CIRCEP.121.010018 34465122

[14] Rivarola EWR Hachul D Chen Wu T Pisani C Scarioti VD Hardy C Long-term outcome of cardiac denervation procedures. JACC Clin Electrophysiol. (2023) 9(8 Pt 1):1344–53. 10.1016/j.jacep.2023.01.032 37558291

[15] Giannino G Braia V Griffith Brookles C Giacobbe F D’Ascenzo F Angelini F The intrinsic cardiac nervous system: from pathophysiology to therapeutic implications. Biology (Basel). (2024) 13(2):105. 10.3390/biology13020105 38392323 PMC10887082

[16] Glikson M Nielsen JC Kronborg MB Michowitz Y Auricchio A Barbash IM 2021 ESC guidelines on cardiac pacing and cardiac resynchronization therapy. Eur Heart J. (2021) 42(35):3427–520. 10.1093/eurheartj/ehab364 34455430

